# Long‐term response to crizotinib in a 17‐year‐old boy with treatment‐naïve ALK‐positive non‐small‐cell lung cancer

**DOI:** 10.1002/cnr2.1483

**Published:** 2022-01-28

**Authors:** Giacomina Megaro, Evelina Miele, Gian Paolo Spinelli, Iside Alessi, Giada Del Baldo, Raffaele Cozza, Ida Russo, Maria Debora De Pasquale, Maria Giuseppina Cefalo, Paolo Tomà, Andrea Carai, Valentina Di Ruscio, Maria Antonietta De Ioris, Angela Mastronuzzi

**Affiliations:** ^1^ Department of Hematology/Oncology, Cell and Gene Therapy IRCCS Bambino Gesù Children's Hospital Rome Italy; ^2^ UOC Oncologia Universitaria, ASL Latina (distretto Aprilia) Sapienza University of Rome‐ Aprilia Latina Italy; ^3^ Department of Imaging Bambino Gesù Children's Hospital, IRCCS Rome Italy; ^4^ Neurosurgery Unit, Department of Neuroscience Bambino Gesù Children's Hospital, IRCCS Rome Italy

**Keywords:** adenocarcinoma, adolescence, ALK, crizotinib, lung

## Abstract

**Background:**

Lung cancer is the leading cause of cancer‐related death. NSCLC accounts for 80–90% of cases. In young patients, adenocarcinoma is the most frequent histotype and 3–7% expresses the rearrangement of ALK oncogene, sensitive to TKIs. Crizotinib is the first ALK inhibitor approved by the FDA.

**Case:**

We present a case of a 17‐year‐old male with metastatic treatment‐naïve ALK‐positive adenocarcinoma. He was treated with crizotinib and obtained a prolonged response with PFS of 33 months.

**Conclusion:**

Crizotinib can be extremely effective in adolescents with treatment‐naïve ALK‐positive NSCLC but fail to prevent a central nervous system relapse. Resistance mechanisms need to be investigated.

## 1 INTRODUCTION

Lung cancer is the leading cause of cancer‐related death representing the first cause in men and the third cause in women, while tobacco smoke is the main risk factor (85% of cases).^1^, ^2^, ^3^

The incidence of primary lung cancer in children is rare, approximately 0.2% of all childhood malignancies.^4^

In adulthood, over 95% of lung cancers are small cell lung cancer (SCLC) and non‐small cell lung cancer (NSCLC).^5^, ^6^ Adenocarcinoma is the most represented in young patients^7^, ^8^, ^9^ with overlapping clinical presentation i with adults (cough, chest pain) but not smoking‐related. The diagnosis is often delayed with more than half of the cases with metastatic spread.^10^, ^11^

In the last 15 years, molecular studies have highlighted the role e of genes called “Oncodrivers” involved in tumor genesis, particularly Epidermal Growth Factor Receptor (EGFR) and Anaplastic Lymphoma Kinase (ALK), which have become the target of specific and selective drugs.^12^

The 10–15% of the Caucasian population expresses activating mutations of EGFR and 3–7% expresses rearrangement of the ALK oncogene; the mutation expression predicts TKIs response.

Crizotinib is one of the first ALK inhibitors approved by the Food and Drug Administration (FDA).^13^ Next‐generation ALK inhibitors such as alectinib and ceritinib were approved for the treatment of ALK‐positive NSCLC patients.^14^, ^15^, ^16^, ^17^, ^18^, ^19^

ALK mutations were reported 11% of adenocarcinoma in younger patients and in only 2–7% of adults.^20^, ^21^, ^22^

There are no specific data about drugs management in pediatric population affected by lung adenocarcinoma and ALK mutation.

We present a case of a seventeen‐year‐old male with naïve ALK‐positive NSCLC and treated with crizotinib. To our knowledge, this is the fifth report of a pediatric case treated with ALK‐inhibitors, the first experiencing a prolonged response and progression‐free survival (PFS).

We extensively reviewed the current literature about ALK positive NSCLC in childhood and the tailored management in pediatric age.

## 2 CASE

**FIGURE 1:**
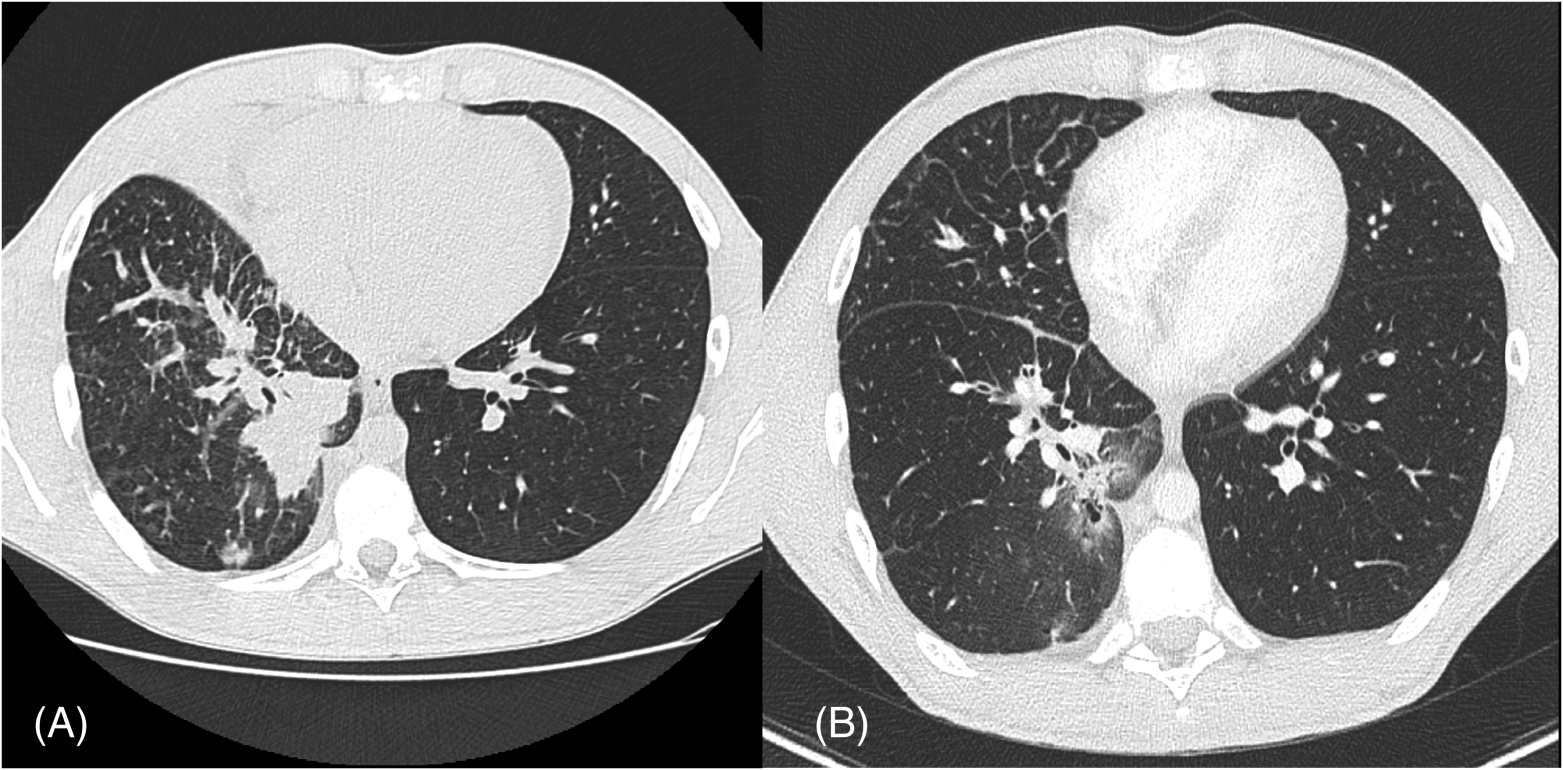
CT scan of the lungs. Adenocarcinoma with consolidation, ground‐glass opacity, centrilobular nodules, interlobular septal thickenin, before treatment (A) and after 2 months of Crizotinib (B)

**FIGURE 2:**
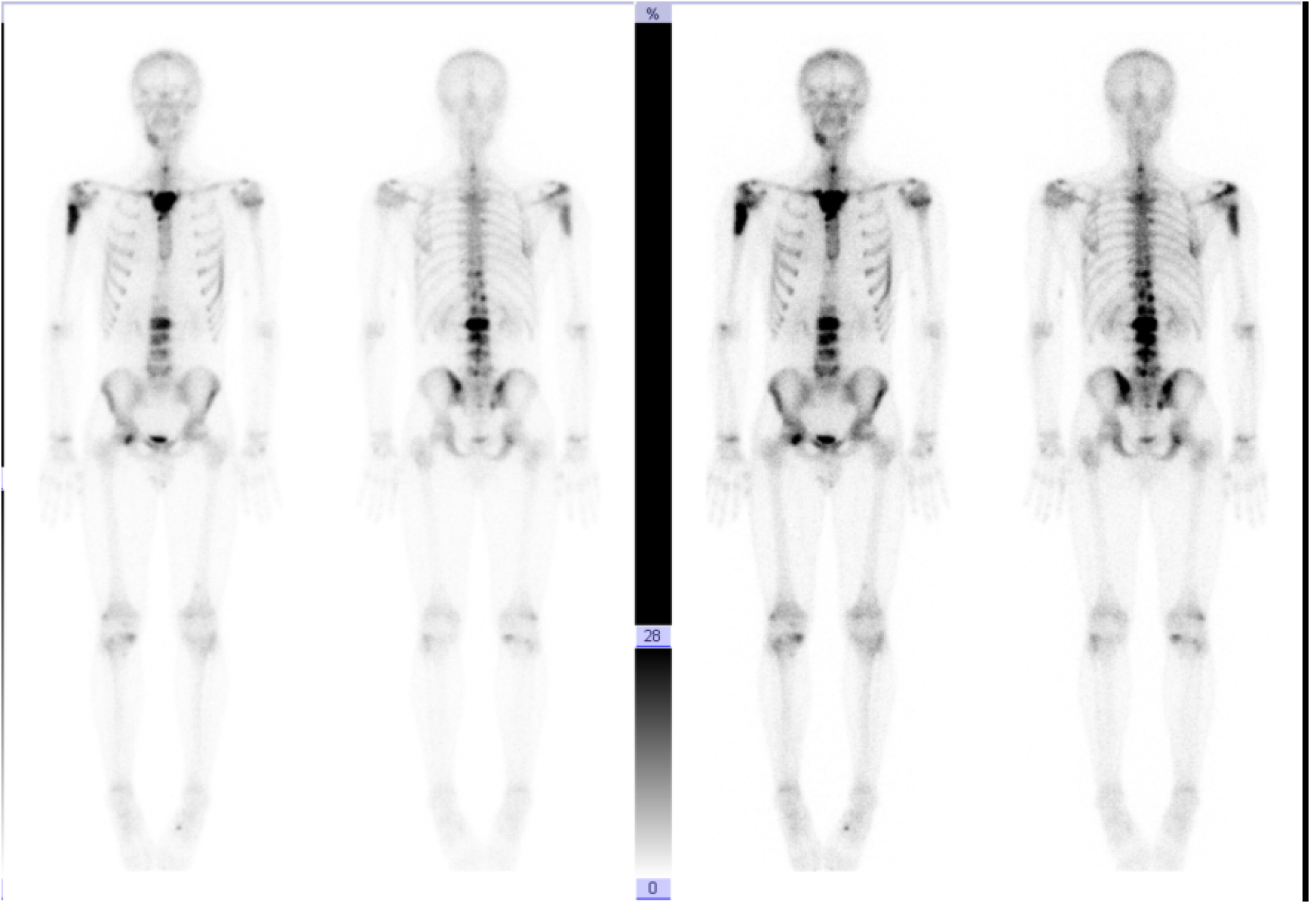
Bone Scintigraphy showing multiple bone metastases at time of diagnosis

**FIGURE 3:**
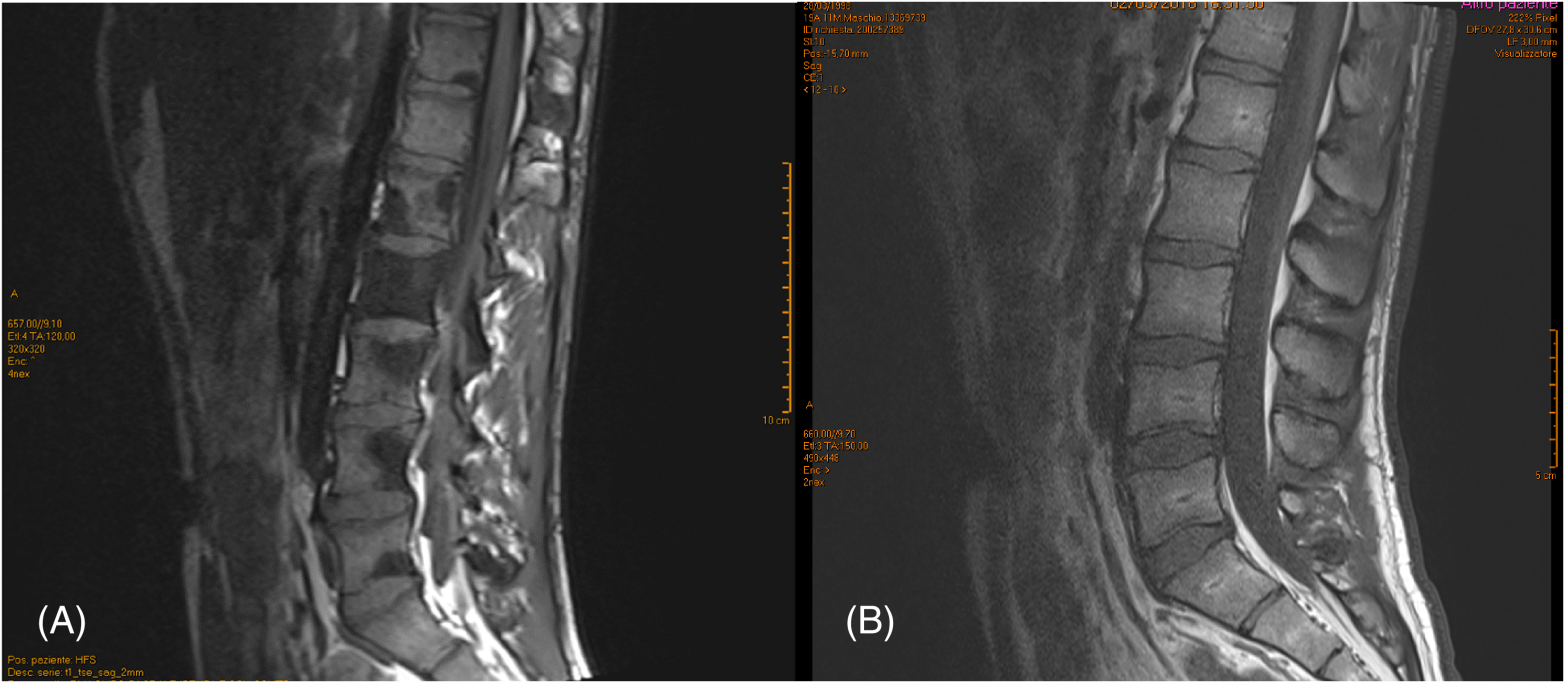
Spinal MRI showing vertebral bone metastase at diagnosis (A) and after 30 months of treatment with Crizotinib (B)

A diabetic, non‐smoker, 17‐year‐old boy was admitted in July 2015 for cough and dyspnea. A chest x‐ray showed bilateral lung nodules confirmed at Computed Tomography (CT)‐scan with lymphadenopathies (Figure 1(A)). Liver, brain and bone metastases were also evident (Figures 2 and 3(A)) .

The supraclavicular node biopsy showed a metastases from pulmonary adenocarcinoma, Cytokeratin 7+ (CK 7+), Transcriptional Thyroid Factor 1+ (TTF1+), p63+, ALK‐rearranged. EGFR and c‐Ros Oncogene 1 (ROS1) wild type.

By analyzing tissue using FISH probe “ON ALK (2q23) Break” (repeat‐free POSEIDON ALK Break Probe, Kreatech, Amsterdam, the Netherlands), ALK rearrangement was detected while no ROS1 translocation was found with the FISH probe “ON ROS1 (6q22) Break” (Repeat‐free POSEIDON ROS1 Dual Color Break Apart Probe, Kreatech, Amsterdam, the Netherlands).

After ALK rearrangement identification, the patient started therapy with crizotinib 500 mg/day with a partial response achieved after 2 months involving lungs (Figure 1(B)), nodes, liver and bone lesions,‐ with a 33 month progression free survival (PFS). (Figure 3(B)).The treatment was well tolerated without grade 3 or 4 toxicity(according to Common Terminology Criteria for Adverse Events (CTCAE) v5.0.

After 33 months of treatment with crizotinib, the patient underwent brain CT scan due to headache. A right fronto‐parietal lesion of approximately 35 × 31 × 30 mm was detected while a smaller lesion was detected on the right lateral ventricle (about 10 mm). Cerebellar tonsils were at the limit of foramen magnum.

Excision of the fronto‐parietal lesion was performed, and histology confirmed a metastasis from ALK‐rearranged pulmonary adenocarcinoma. Radiotherapy was then performed at 3000 cGy on residual tumor and 850 cGy on right paraventricular posterior lesion.

After 2 months of treatment, a chest and abdomen CT‐scan showed pulmonary stability and partial response of the hepatic lesions.

Liver metastases rapidly progressed after 7 months of alectinib and the patient died of liver failure.

## 3 DISCUSSION

Lung lesions and thoracic lymphadenopathies in adolescents and young adults (AYA), raises suspicion of lymphoma. Hodgkin and non‐Hodgkin lymphoma represent, respectively, 27% and 24% of all cancers in the 15–19 year and 20‐to‐30‐year age groups^23^ while other histologies are infrequent. We present a case of ALK translocated adenocarcinoma in AYA with prolonged PFS.

According to our knowledge, this is the fifth case reported of adolescent with diagnosis of pulmonary ALK‐translocated adenocarcinoma^24^, ^25^, ^26^ and the third treated with crizotinib as first‐line treatment.^24^, ^25^ (Table 1).

**TABLE 1:** Case reports‐ ALK‐positive lung adenocarcinoma in adolescents

Author	Age/gender	TNM stage	First line treatment	Outcome
Balzer 2018	14/F	IV	Alectinib	Alive with controlled disease (9 months)
Usmani 2018	18/M	IV	Alectinib	Alive with controlled disease (8 months)
Scarpino 2016	17/M	IV	Crizotinib	Died from disease (7 months)
Kim 2012	14/F	IV	Crizotinib	Alive with controlled disease (16 months)

At the time of diagnosis, the crizotinib represented the first‐line treatment available.

At onset, he received crizotinib and obtained at least a partial response to all lesions after 3 months, demonstrating high sensitivity to tyrosine kinase inhibition and confirming the best response to crizotinib by 12 weeks as reported.^26^

A 33 month PFS is impressive considering the PROFILE1014 trial results that showed a 10,9 months median PFS.^27^

The treatment was well tolerated by our patient, experiencing only grade 1 neutropenia and grade 1/grade 2 hypertransaminasemia.

In our case, crizotinib demonstrated good control of extra‐Central Nervous System (CNS) disease but limited control on brain progression, supporting the poor penetration of the blood–brain barrier (BBB).^28^, ^29^, ^30^

Surgery was performed on our patient after CNS dissemination as well as radiotherapy and a second‐line therapy with alectinib. As demonstrated by the ALEX trial,^31^ alectinib was significantly superior to crizotinib in controlling brain progression.

The permeability of the BBB from alectinib and the results obtained on the control of brain metastases^31^ limited the role of radiotherapy and surgery in this setting, particularly in untreated patients.

In our case, we decided to treat the frontal symptomatic brain lesion. and to add radiotherapy considering the post‐ surgical residual tumor and paraventricular lesion. We then started alectinib as second‐line TKI.

Our report suggests that crizotinib can be extremely effective in ALK‐positive lung adenocarcinoma without CNS involvement as a first line treatment in AYA patients. The occurrence of isolated CNS relapse, highlights the need for new TKI sequence studies to optimize the management of ALK‐translocated lung cancer.

The optimal sequence of TKI in ALK‐positive lung cancer and the role of surgery and radiotherapy at CNS dissemination remain critical. Resistance mechanisms to TKIs need to be further investigated for a better management of these cases in childhood and adolescence.

## CONFLICT OF INTEREST

No potential sources of conflict of interest.

## AUTHOR CONTRIBUTIONS

**Giacomina Megaro:** Conceptualization; investigation; writing‐review & editing. **Evelina Miele:** Conceptualization; formal analysis; methodology. **Gian Paolo Spinelli:** Supervision. **Iside Alessi:** Writing‐review & editing. **Giada del Baldo:** Resources. **Raffaele Cozza:** Data curation. **Ida Russo:** Conceptualization; resources. **Maria Debora de Pasquale:** Data curation; formal analysis. **Maria Giuseppina Cefalo:** Data curation; methodology. **Paolo Tomà:** Data curation; methodology; software. **Andrea Carai:** Supervision. **Valentina di Ruscio:** Visualization. **Maria Antonietta de Ioris:** Writing‐review & editing. **Angela Mastronuzzi:** Conceptualization; supervision; writing‐original draft; writing‐review & editing.

## ETHICAL STATEMENT

Institutional approval was not required for a case report. All the patient information was de‐identified for the purpose of this case report. Patient consent was therefore not obtained for publication.

## Data Availability

Data will be made available upon reasonable request.
